# Role of husband’s attitude towards the usage of contraceptives for unmet need of family planning among married women of reproductive age in Pakistan

**DOI:** 10.1186/s12905-021-01314-4

**Published:** 2021-04-19

**Authors:** Muhammad Farhan Asif, Zahid Pervaiz, Jawad Rahim Afridi, Ghulam Abid, Zohra S. Lassi

**Affiliations:** 1 National College of Business Administration and Economics, Lahore, Pakistan; 2 University of Peshawar, Peshawar, Pakistan; 3 Department of Business Studies, Kinnaird College for Women, Lahore, Pakistan; 4 Robinson Research Institute, The University of Adelaide, Adelaide, Australia

**Keywords:** Husband’s attitude, Unmet need, Pakistan

## Abstract

**Background:**

Family planning services deliver a wide range of benefits to the well-being of females and the community. It can curtail the risk of maternal and neonatal mortality through the reduction in abortions and pregnancies. The government of Pakistan has been struggling to convince people about the usefulness of family planning programs. However, different factors related to social norms, values, and culture are important to determine the success of these programs. One such factor is the patriarchal structure of Pakistani society where most of the household decisions are made by men. The objective of this research is to examine the role of the husband’s attitude towards the usage of contraceptives for the unmet need of family planning (UMNFP) among married women of reproductive age (MWRA) in Pakistan.

**Method:**

The dataset of Pakistan Demographic and Health Survey 2017–18 is utilized to examine the role of the husband’s attitude towards the usage of contraceptives in UMNFP among MWRA in Pakistan.

**Results:**

The UMNFP was considerably lower among MWRA between 40 years and above compared to women 15–19 years. The odds of UMNFP were higher among women and men who were educated up to the primary level compared to those with no education. Odds of UMNFP were higher among women from the poor wealth quintile compared to the poorest wealth quintile; similarly, it was significantly lower among women who were from the richer and the richest wealth quintile compared to the poorest wealth quintile. The odds of UMNFP were lower among women who were employed compared to those who were not employed. Lastly, the odds of UMNFP were higher among women whose husbands opposed to using contraceptives, who perceived that there was a religious prohibition for such use and when a decision on the contraception use was solely made by the husband.

**Conclusions:**

Husband’s attitude towards the usage of contraceptives is an important predictor of UMNFP. Liaising with the community and religious leaders to persuade people particularly men about the usefulness of family planning programs and encouraging men to understand their women’s say in using contraceptives should be encouraged.

## Background

Unmet need for family planning (UMNFP) is described as “the proportion of married women of reproductive age (MWRA) who are not using any method but would like to postpone the next pregnancy (unmet need for spacing), or who do not want any more children (unmet need for limiting)” [1, 2].

Family Planning (FP) services deliver a wide range of benefits to the well-being of females and the community. It can curtail maternal and neonatal mortality through a falling number of abortions and pregnancies [3]. Reproductive health and FP programs significantly contribute to falling fertility and improving parental and child health in the less developed countries [1, 4–6]. UMNFP is the main cause behind childbearing at a very young age and closely spaced births [7–10], and often been found associated with physical abuse, unsafe abortion, and poor maternal health [11].

UMNFP occurs due to several reasons which include fewer choices of FP methods, lack of access to contraception, unaffordability, fear or experience of having side effects, religious and cultural opposition, men’s dominance in decisions for using FP, and poor quality of available FP services [12].

The economic and social development of communities and countries determine the level of contraceptives and reasons for UMNFP [13]. Numerous health attributes of a population, economic and population policy of environments, individual attributes, and household formation were linked to contraceptive [14]. A number of FP methods available, cost of FP methods and fee for service, women’s participation, and their socio-demographic and economic status, medical boundaries and misinterpretation have been reported as determinants of UMNFP [15]. Furthermore, a woman and her partner’s education, knowledge about FP methods, access to media, support of family members to use contraceptives, migration status, age, parity, wealth status, place of residence, the experience of abortion, religious constraint are linked with the use of FP methods [14, 16, 17].

UMNFP is found to be higher among younger females who have lived in rural areas, who have fear of side effects, and who do not know contraceptive methods or their availability [3, 18, 19]. It is also noticed that UMNFP is significantly lower among women who are educated, whose husband is educated as well, already have other living children, and belong to high socioeconomic status [20–23].

UMNFP also occurs because the providers do not offer adequate information as well as any motivation regarding FP usage [24]. The involvement of the community and religious leaders can play an important role in the success of FP programs [25]. Males are held responsible for most of the reproductive decisions of a female particularly in low- and middle-income countries (LMICs) [26, 27]. When the decision-making of contraception usage is shared between males and females, it results in an effective continuation of contraceptive methods [26]. Studies have shown that when the decision-making for FP is mutual the use of contraceptives is greater [28, 29]. The dominant and important role of men in household decision-making regarding female employment, contraceptive use, and the reproductive decision has been persistently recognized by the global community [30–32]. Evidence from the literature shows that female preferences and behavior about reproduction and fertility are significantly influenced by their husband’s reproductive stimulus. This is due to the female’s financial and social dependency on the husband [33]. Such dependency on women is the result of traditional beliefs that women are subordinate to their husbands [31, 34]. In LMICs female dependency on men has been more crucial [35].

Effective FP programs can work to reduce UMNFP [36]. There is a huge gap found between current users and the total demand of FP [37]. In LMICs, 225 million women of reproductive ages would want to delay or stop bearing a child but are not using any FP method [12]. In Eastern Europe, Asia, and North Africa, around 45% of women are facing an issue of UMNFP [38]. Overall, from the current years, UMNFP is approximately 17% in Pakistan [39], 24% in Nepal [40], 22% in Ethiopia [41], 22% in Tanzania [42], 30% in Guyana [12], 32% in Peru [43], 11% in Indonesia [44], 12% in Bangladesh [45] and 13% in India [46]. The high prevalence of UMNFP is a major problem of less developed countries and therefore sympathetic causes and causal determinants of UMNFP would help in reducing the prevalence of UMNFP. In Pakistan, although the contraceptive uptake has been increasing by 1% per year; the rate of UMNFP is still high and over 56% of the married female of fertile age intend to use FP services and only 39% use them. Although the research has underscored the importance of UMNFP, it has not been completely associated with the role a husband plays in uptake on contraceptives in a nationally representative sample.

Pakistan is an LMIC with a strong patriarchal structure where men play a vital role in decision-making regarding family affairs especially reproduction. Therefore, it is important to study the role of the husband’s attitude towards the usage of contraceptives in determining the UMNFP among married women in Pakistan. Such role has been rarely studied in the case of Pakistan probably due to the reason that past demographic surveys complied limited information about men’s attitude towards the usage of FP services.

## Methods

### Data source

The Pakistan Bureau of Statistics provided the household lists for sampled zones in the country. The sample size based on 16,240 households, out of which 7,980 households were in urban areas and 8260 in rural areas was assessed to provide sensible accuracy for the survey indicators and involved two-stage sampling. At the first stage of sampling, 580 primary sample units (285 in urban areas and 295 in rural areas) were selected using systematic sampling technique and in the second stage of sampling, a static number of 28 households were arbitrarily selected in all cluster by an equal probability systematic sampling procedure. A total of 50,495 married women age 15–49 were interviewed in 2017–18 [39]. After removing participants with missing data, information on 12,113 women was analyzed.

### Measurement

To examine the effects of husband’s opposition on unmet need for FP, the functional form of the model used was:

$${\text{UMNFP}} = {\text{f}}\left( {{\text{W}}.{\text{AGE}},{\text{ W}}.{\text{EDU}},{\text{ WSH}},{\text{ WES}},{\text{ H}}.{\text{EDU}},{\text{ HO}},{\text{RC}},{\text{DC}}} \right)$$

The logistic regression equation for the above functional form of the model can be written as

$${\text{Log}}\left( {{\text{odds}}} \right) = {\text{logit}}\left( P \right) = \ln \left( {P/1{-} \, P} \right)$$

$$\begin{aligned} {\text{UMNFP}} & = {\text{Logit}}\left( p \right) = {\alpha } + b_{1} \left( {{\text{W}}.{\text{AGE}}} \right) + b_{2} \left( {{\text{W}}.{\text{EDU}}} \right) + b_{3} \left( {{\text{WSH}}} \right) \\ & \quad + b_{4} \left( {{\text{WES}}} \right) + b_{5} \left( {{\text{H}}.{\text{EDU}}} \right) + b_{6} \left( {{\text{HO}}} \right) + b_{7} \left( {{\text{RC}}} \right) + b_{8} \left( {{\text{DC}}} \right) + {\varepsilon } \\ \end{aligned}$$

where UMNFP = Unmet need for family planning. The variable has been constructed by extracting information from PDHS. Unmet need for family planning exists when a woman, who wants to use any method of family planning for spacing and limiting the children, does not do so due to any reason. Pakistan Demographic and Health Survey have presented the data on UMNFP. This information has been utilized to construct a dichotomous variable of UMNFP. It is divided into two categories as women having UMNFP (spacing and limiting) and women not having UMNFP.

W.AGE = The age of women has been categorized into seven age groups of five-year group interval i.e. 15–19 and so on.

W.EDU = Education of women is divided into four categories, i.e. no education, primary education, secondary education, and higher education.

WSH = Wealth status of the household. The variable was constructed by using a household asset and residence characteristics. Every household was given a score for all assets and a summation of the score was taken for every household. Each person was ranked as per scores of the households in which they resided. Wealth status was divided into quintiles from poorest to richest [39]. If women belong to a household placed in the first quintile then coded as 1, if women belong to the household of the second quintile then coded as 2, if women reside in a household of the third quintile then coded as 3, if women reside in household belonging to the fourth quintile then coded as 4 and if women reside in household falling in the fifth quintile then coded as 5.

WES = Women’s employment status. The variable has been categorized into two categories, i.e. currently unemployed, and currently employed. Coded as 1 if employed and 0 otherwise.

H.EDU = Husband’s education is divided into four categories, i.e. no education, primary education, secondary education, and higher education.

HO = A question from measuring the husband's opposition is “Would you say that husband’s opposed for not using contraceptive”. It has been categorized into two categories, i.e. husband is opposed to using contraceptives, and the husband is not opposed to using the contraceptive.

RC = Religious constraint is measuring from the question “Would you say that religious prohibition for using contraceptive”. It has been divided into two categories, i.e. religious constraint for using contraceptive and no religious constraint for using a contraceptive.

DC = A question from measuring the decision-maker for not using contraceptives is “Would you say that not using contraception is mainly your decision, mainly your husband's decision”. It is divided into two categories, i.e. women's decision for not using contraceptive and husband's decision for not using a contraceptive.

Descriptive statistics were presented using frequency, percentage, and mean with standard deviation for categorical variables. For our empirical investigation, we have used binary logistic regression as the dependent variable was dichotomous i.e. having UMNFP or not having UMNFP. All analyses were performed in SPSS version 20.

## Results

The socio-economic characteristics of the female are presented in Table 1. Data from 12,113 women were analyzed. Of these, the majority of the women were aged between 45 and 49 years old, and in total more than half of the women were 35 years and older. More than half of all women had no education (66.0%); similarly; one-third of all husbands (36.9%) had received no education. Almost half (51.1%) belonged to either the poor or poorest wealth quintile and 85% of the women were not currently employed. The majority (86%) of the husbands did not oppose contraceptive use and it was not a constraint because of religious reasons (95%). However, almost 86% of the time it was the husband’s decision on contraception use. Overall, half of the women (51.6%) had UMNFP.

**Table 1:** Description of socio-economic characteristics of women

Description of variables	Frequency	Percentage
*Age of women*		
15–19	101	0.8
20–24	606	5.0
25–29	1,590	13.1
30–34	1,889	15.6
35–39	2,621	21.6
40–44	2,511	20.7
45–49	2,795	23.2
*Women’s education*		
No education	7,993	66.0
Primary	1,539	12.6
Secondary	1,690	14.0
Higher	891	7.4
*Wealth status of women’s household*		
Poorest	3,185	26.3
Poorer	2,999	24.8
Middle	2,346	19.3
Richer	1,807	14.9
Richest	1,776	14.7
*Women’s employment status*		
Currently unemployed	10,302	85.0
Currently employed	1,811	15.0
*Husband’s education*		
No education	4,467	36.9
Primary	1,705	14.1
Secondary	3,869	31.9
Higher	2,072	17.1
*Reason not using: husband opposed*		
No opposed	10,458	86.3
Opposed	1,655	13.7
*Reason not using: religious prohibition*		
No prohibition	11,512	95.0
Prohibition	602	5.0
*Decision maker for not using contraceptive*		
Women	1,697	14.0
Husband	10,416	86.0
*Unmet need for family planning*		
No	5,863	48.4
Yes	6,250	51.6

The results of binary logistic regression are presented in Table 2. The UMNFP was significantly lower among women between 40 years and above compared to women 15–19 years. The odds of UMNFP were higher among women and men who were educated up to the primary level compared to those with no education. Odds of UMNFP were higher among women from the poor wealth quintile compared to the poorest wealth quintile; similarly, it was significantly lower among women who were from the richer and the richest wealth quintile compared to the poorest wealth quintile. The odds of UMNFP were lower among women who were employed compared to those who were not employed. Lastly, the odds of UMNFP were higher among women with husbands who opposed the use of contraception, the women who perceived that there was a religious prohibition for such use and when a decision on the use was solely made by the husband. The results indicate that religious prohibition of the use of contraception as well as husbands' opinion about the use were important factors to determine UMNFP. However, a comparison of the odds of UMNFP from both of these variables indicates that the effects of husbands' opinions toward contraception are larger than the effects of religious prohibition.

**Table 2:** Results of binary logistic regression

	B	Sig	Odds ratio	95% C.I. for odd	
				Lower	Upper
Constant	.271	.242	1.311	–	–
*Age of women*					
15–19	*Reference*				
20–24	−.170	.434	.843	.550	1.292
25–29	.013	.950	1.013	.673	1.524
30–34	.145	.484	1.156	.770	1.736
35–39	−.072	.726	.930	.622	1.393
40–44	−.711	.001	.491	.328	.735
45–49	−1.130	.000	.323	.216	.484
*Women’s education*					
No education	*Reference*				
Primary	.153	.012	1.165	1.035	1.312
Secondary	.121	.052	1.128	.999	1.275
Higher	.082	.001	1.026	.916	1.575
*Wealth status of women’s household*					
Poorest	*Reference*				
Poorer	.141	.008	1.151	1.037	1.277
Middle	-.063	.283	.939	.837	1.053
Richer	−.305	.000	.737	.648	.839
Richest	−.391	.000	.677	.584	.783
*Women’s employment status*					
Currently unemployed	*Reference*				
Currently employed	−.107	.048	.899	.809	.999
*Husband’s education*					
No Education	*Reference*				
Primary	.145	.016	1.156	1.028	1.300
Secondary	.094	.628	1.105	.929	1.130
Higher	.082	.212	1.085	.954	1.235
*Reason not using: husband opposed*					
No opposed	*Reference*				
Opposed	.853	.000	2.347	2.087	2.639
*Reason not using: religious prohibition*					
No prohibition	*Reference*				
Prohibition	.151	.089	1.163	.977	1.383
*Decision maker for not using contraceptive*					
Women	*Reference*				
Husband	.290	.000	1.336	1.215	1.469

## Discussion

Both husband’s attitudes towards the usage of contraceptives and religious constraints are the important indicators that affect UMNFP. Women with husbands who opposed and religious prohibition to use FP are more likely to have UMNFP. Husband’s opposed to using FP is also a strong predictor of UMNFP. It is the responsibility of the community and religious leaders to address their community regarding the use of FP. UMNFP is higher in those women whose less participate for the decision to use FP as compared to husband. It is concluded that men and women can use contraception effectively if they discuss it more often and will probable to have fewer children. Husband plays a critical role in the decision-making regarding the use of contraception, especially in the early years of marriage.

Women’s age and the level of unmet need are comprehensibly associated with each other. Unmet need is related to spacing births at a young age for women whereas in old age it is to limit births. Unmet need, typically for most women, rises to the top till the late thirties and declines in forties. The possibility of UMNFP is highest among women of age early thirties, but it is proved insignificant statistically. UMNFP declines with the increase in age. As most of the women attain the desired number of children till the age of 35–39, they will be likely to use contraceptives. Therefore UMNFP is highest among women belongs to the middle age group. On the other, for postponing their next pregnancy, the tendency of young women to space is expected to be higher. Our results stand consistent with the results of previous studies [18, 21, 47–50].

The tendency of UMNFP is less in educated women than uneducated women. It is because of the higher level of awareness that educated women have access to FP services. In the same way, educated women are more empowered in decision-making for the use of contraceptives. Our results are aligned with earlier studies [18, 21, 51, 52]. The chances of UMNFP are less for women of wealthier families as compared to those who belong to poorer families. Because wealthy women are capable to access modern contraceptives in a better way than poor women [1]. The odds ratio indicates that currently employed women are less probable to have UMNFP as compared to currently unemployed women. Moreover, women’s employment is also found to be associated with their empowerment and hence they can be in a better position to decide on the use of contraceptives [53]. Similarly, the husband’s education also plays a vital role in the reduction of UMNFP, as it promotes mutual decision making and [1, 54, 55].

The results are extracted from the data provided by the women respondents belong to the age group of 15–49. The perception of the respondents regarding the family planning service, related views, and socioeconomic characteristics laid vital influence on the family planning methods, its cost and availability issue. Specifically, In the context of Pakistan the mentioned fact can be considered as reality in LMICs. Further empirical support will be useful to establish the significance of the prevailing factors of the UMNFP. Furthermore, there is a need to investigate the reasons for region base differences of wide UMNFP.

## Conclusions

The study has identified that women’s age, education, their employment status, wealth index, husband’s education, husband's attitude towards the usage of contraceptives, religious prohibition, and husband's decision on contraception are significantly associated with UMNFP. Understanding these determinants is essential for planning and executing behavior change exercises to underscore the importance of implementing gender-transformative interventions and to influence FP choice among couples, particularly in societies where socio-cultural norms pre-dominantly exist. In light of the results from this paper, national policies in Pakistan should also consider integrating FP programs with education, employment, and media sectors. It important that government takes measures to improve the educational and literacy status of the population particularly girls; which will bring about changes in the employment and economic status of the population. Creating employment opportunities for women with effective public policies, and effective use of media in raising awareness can all bring a positive change in society which can also impact on improving UMNFP. Liaising with the community and religious leaders to persuade people particularly men about the usefulness of FP programs and encouraging men to understand their women’s say in using contraceptives should be encouraged.

## Data Availability

We have used the secondary data of PDHS 2017–18. Available at: https://www.nips.org.pk/PDHS_Data_Set.htm.

## Abbreviations

FP: Family planning
PDHS: Pakistan Demographic and Health Survey
UMNFP: Unmet need for family planning
LMICs: Low- and middle-income countries
MWRA: Married women of reproductive age
